# HLA-F regulates the proliferation of trophoblast via PKM2-dependent glycolysis in the pathogenesis of preeclampsia

**DOI:** 10.1186/s10020-025-01201-w

**Published:** 2025-04-18

**Authors:** Ruiling Xu, Yu Huang, Wenchi Xie, Dan Luo, Jie Mei, Xinghui Liu, Fulin Liu, Fangyuan Luo

**Affiliations:** 1 Department of Obstetrics and Gynecology, Sichuan Provincial People’s Hospital, Sichuan Academy of Medical Sciences, University of Electronic Science and Technology, Chengdu, China; 2 Department of Obstetrics and Gynecology, The Affiliated Hospital of Southwest Medical University, Luzhou, Sichuan China; 3 Department of Obstetrics and Gynecology, West China Second University Hospital of Sichuan University, Chengdu, China; 4 Sichuan Provincial Key Laboratory for Human Disease Gene Study, Center for Medical Genetics, Department of Laboratory Medicine, Sichuan Provincial People’s Hospital, Sichuan Academy of Medical Sciences, University of Electronic Science and Technology, Chengdu, China

**Keywords:** Preeclampsia, HLA-F, Trophoblast, Proliferation, PKM2, Lactylation

## Abstract

**Background:**

The regulatory molecule Human Leukocyte Antigen F (HLA-F) has been implicated in trophoblast proliferation during pregnancy, and reduced levels of this antigen have been identified in trophoblast cells of patients with preeclampsia. This study aimed to analyze the effect and mechanism of HLA-F on the proliferation of trophoblast and the underlying mechanism of reduced HLA-F involved in preeclampsia.

**Methods:**

q-PCR, Western blot (WB), and Immunohistochemistry (IHC) were used to detect the expression of HLA-F and Pyruvate Kinase Muscle isoform 2 (PKM2) in placenta tissues. Jar cells were transfected with overexpression lentivirus, specific siRNA, and shRNA to regulate corresponding genes. Immunofluorescence was used to analyze the expression and distribution of HLA-F and PKM2. Extracellular and intracellular lactate, pyruvate, and enzymatic activity of PKM2 were measured using the corresponding assay kits. Cell proliferation was measured by CCK8, MTT, colony formation assay, and Mini patient-derived xenograft (Mini-PDX). Chromatin Immunoprecipitation and deep sequencing (ChIP-seq) and 4-dimensional label-free quantitative proteomics (4D-LFQP-LA) were used to analyze the HLA-F-binding DNA sequences and the differential lactylation proteins in HLA-F-overexpression Jar and its control.

**Results:**

The expression of HLA-F is reduced in extravillous trophoblast and villous cytotrophoblast from patients with preeclampsia. Over-expression of HLA-F promoted proliferation while under-expression inhibited it. Further experiments demonstrated that over-expression of HLA-F promoted expression of the PKM2 protein and its enzymatic activity, resulting in enhanced glycolysis in Jar cells. Specifically, we determined that HLA-F regulated the expression of PKM2 by binding the promoter of *PKM*, and promoted PKM2 enzyme activity by down-regulating the lactylation of residue K305. Moreover, silencing PKM2 with siRNA reduced HLA-F–mediated glycolysis and proliferation in HLA-F–overexpressing Jar cells. Finally, we corroborated these results using a MiniPDX model, with which we confirmed that the PKM2 agonist TEPP-46 promoted the proliferation of ShHLA-F Jar cells.

**Conclusions:**

The reduced expression of HLA-F in placental trophoblast cells resulted in the downregulation of both PKM2 transcription and protein expression. Concurrently, the relative upregulation of lactylation at PKM2 K305 contributed to a decline in enzyme activity, further exacerbating glycolysis dysfunction. Collectively, these alterations led to a suppression of trophoblast proliferation capacity and involvement in the pathogenesis of preeclampsia.

**Graphical Abstract:**

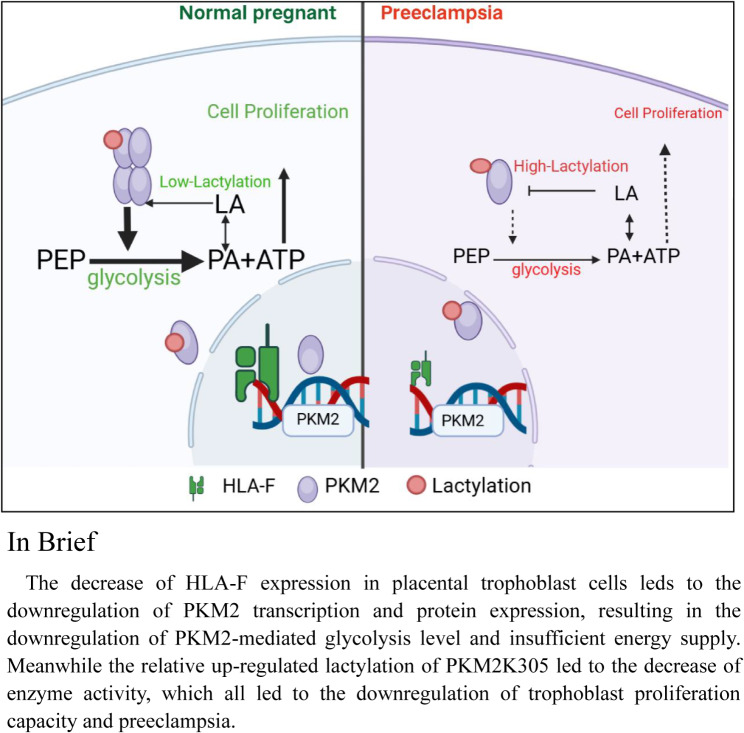

**Supplementary Information:**

The online version contains supplementary material available at 10.1186/s10020-025-01201-w.

## Introduction

Human Leukocyte Antigen F (HLA-F), a nonclassical HLA class I molecule, is extensively expressed by decidua stromal cells, villus cytotrophoblast (VCTs), extravillous trophoblast (EVTs), and immune cells at maternal-fetal interface (Luo et al. 2023). It can form major histocompatibility complexes (MHCs) with β_2_M and peptides to present antigens to immune cells (Sim and Sun 2017). The recognized receptors for HLA-F include KIR3DS1/L1/L2 on natural killer (NK) cells (Lunemann et al. 2018), LILRB1/2 on monocytes/macrophages and a recently identified inhibitory receptor, VSIR, expressed on EVTs, NK cells, and monocytes/macrophages (Katabathula et al. 2022). Through ligand-receptor interaction, HLA-F-expressing cells can modulate immune cell functions and promote maternal-fetal immune tolerance. Additionally, HLA-F can exist as an open conformer (OC) devoid of peptides and β_2_M (Sim and Sun 2017). These OCs of HLA-F are distributed in the extracellular matrix and the nucleus (Luo et al. 2023); However their biological roles remain largely unexplored. In a previous study, we detected reduced levels of HLA-F in EVT cells of patients with preeclampsia, a pregnancy-specific multi-etiological disorder characterized by maternal hypertension and organ damage. Moreover, the results of cell viability experiments suggested that HLA-F might promote trophoblast proliferation (Luo et al. 2023). Similarly, via immunohistochemical analysis of placenta decidua, Dunk and colleagues also found that HLA-F protein expression was decreased in the EVT cells of patients with preeclampsia (Dunk et al. 2022). To date, though, the mechanism by which HLA-F reduction might contribute to the pathogenesis of preeclampsia remains unknown.

In addition to immune dysregulation, another potential mechanism linking reduced HLA-F expression to preeclampsia might involve its impact on glycolysis. In a glioma cell line, HLA-F was found to promote cell proliferation in vitro by promoting HK2 protein stabilization and glycolysis (Chen et al. 2021b). Inhibition of glycolysis was reported to reduce the migration and invasion of trophoblast (Liang et al. 2023), and reduced glycolysis in trophoblast tissues has been linked to both recurrent abortion (Xiong et al. 2024; Liang et al. 2022), and preeclampsia (Song L 2024; Yang et al. 2022; Bloxam et al. 1987). Glycolysis—especially aerobic glycolysis, which produces substantial amounts of pyruvate, lactate, and preliminary energy—is crucial for establishing a favorable micro-environment for embryo implantation and trophoblast migration. Indeed, scRNA-seq analysis of first-trimester decidua revealed that the dominant NKG2C + NK population had a higher level of glycolysis (Vento-Tormo et al. 2018), while another scRNA-seq study reported decreased glycolysis in decidua from patients with late-onset preeclampsia (Yang et al. 2023). In our previous study, we found that high levels of HLA-F expression in NK cells promoted NKG2C + NK cell expansion and that reducing HLA-F expression in trophoblast inhibited its proliferation and migration (Luo et al. 2023). However, that study did not specifically investigate how decreased HLA-F expression in EVT cells might affect glycolysis, and how this might influence other cell functions such as proliferation.

Glycolysis is characterized by three key rate-limiting enzymes, of which one, pyruvate kinase, can be found in four isoforms (M1, M2, L, and R) in mammals (Alquraishi et al. 2019). PKM1 is mainly expressed in energy-demanding muscle and brain cells, while PKM2 is mainly expressed in embryonic cells, stem cells, and tumor cells with strong anabolic demand. Unlike enzymatically active PKM1, PKM2 mainly exists in monomeric or dimeric forms and is subject to complex allosteric regulation that directs its pyruvate kinase activity (Zheng et al. 2021). It has been reported that lactate, a product of glycolysis, can increase the cytoplasmic distribution—and thus the enzymatic activity—of PKM2 in LPS-incubated macrophages (Wang et al. 2022). In addition to the relative effects of cytoplasmic or nucleolar distribution, the activity of this enzyme is also influenced by post-translational modifications (PTMs) that alter its structure and function, and these play a key role in the endogenously allosteric regulation of PKM2 (Prakasam et al. 2018). For example, citrullination of the R106 residue (Coassolo et al. 2021) and acetylation at residue K433 promote its activity (Das Gupta et al. 2020), while O-GlcNAcylation or K311 succinylation inhibit the catalytic activity of this enzyme (Singh et al. 2019). Recent research has found that lactate can regulate PKM2’s pyruvate kinase activity through a novel PTM of PKM2: lysine lactylation (Wang et al. 2022).

Given the close relationship between glycolysis and the proliferative capacity of trophoblasts, as well as the influence of key enzymes such as PKM2 on this process, we hypothesize that HLA-F may promote trophoblast proliferation by modulating glycolysis. Furthermore, lactate, a byproduct of glycolysis, is likely to regulate the activity of key glycolytic enzymes through lactylation. To address this, the current study utilized a comprehensive approach, including chromatin immunoprecipitation sequencing (ChIP-seq), 4-dimensional label-free quantitative proteomics integrated with lactylation analysis (4D-LFQP-LA), and a Mini patient-derived xenograft (MiniPDX) model, to explore the regulatory mechanisms through which reduced HLA-F expression inhibits trophoblast proliferation and glycolysis, ultimately contributing to the pathogenesis of preeclampsia. For the first time, we identified the K305 residue of PKM2 as a site modified by lactate. We provide evidence that HLA-F can induce the protein expression of PKM2 and inhibit the lactylation of residue K305. This increases the pyruvate kinase activity and cytoplasmic distribution of PKM2, thereby promoting glycolysis and the proliferation of trophoblast. This study may offer novel insights into the mechanisms underlying preeclampsia development and shed light on the metabolic regulatory functions of the classical antigen-presenting molecule HLA-F.

## Materials and methods

### Ethics and collection of human placentas

The collection of human placentas was approved by the Ethics Committee of Sichuan Provincial People’s Hospital (No. 2022 − 311) with informed written consent from patients. A total of 24 cases, 12 cases with preeclampsia and 12 cases with normal pregnancies, of the placenta were obtained from the term according to the power analysis.

### Immunohistochemistry

Immunohistochemical (IHC) staining was strictly performed according to previous study(Hackmon et al. 2017). Rabbit anti-human HLA-F (Bioss, bs-17544R/, 1:100) was overnight incubated and visualized (Dako Cytomation, Denmark). The 3,3’-diaminobenzidine (DAB) intensity of three proximal EVT cells (close to the decidua surface) was measured on digital images by 2 independent investigators blinded to the patients group using Image J software (National Institutes of Health, USA) and based on the %Area value of the selected EVTs.

### Western blot analysis

Western blot analysis was performed as described previously (Luo et al. 2023). The primary antibodies used included Anti-HLA-F (Bioss #bs-17544R, 1:2000), -HK2 (Abcam #ab209847, 1:1000), -PKM2 (Abcam #ab150377, 1:5000), -Flag (Bioss #bsm-33346 M,1:5000), -GAPDH (Abclonal #AC033,1:200000) and -β-actin (Abclonal #AC026, 1:50000).

### RT-PCR

Total RNA was isolated from cells using Trizol and cDNA was synthesized using the Molpure^®^ Cell/Tissue Total RNA Kit (Yeasen, #19221ES50). The cDNA samples were subjected to a real-time quantitative polymerase chain reaction (RT-qPCR) using the TB Green TM Premix Ex TaqTM ii (Tli RNaseH Plus) (Takara, RR820A) performed on a QuantStudio TM3 PCR instrument (Bio Tek). All procedures were performed according to the manufacturer’s protocols. The primer sequences are listed in Table S1.

### Cells and plasmid transfection

The human choriocarcinoma Jar and BeWo cell lines and human trophoblast HTR-8/SVneo cell lines were commercially obtained from Procell (Wuhan, China). The cells were cultured in DMEM containing 10% FBS (SH30084.03, HyClone Fetal Bovine Serum, Characterized, Australian origin; HyClone) and 1% double antibiotic (100 mg/L streptomycin and 100 U/mL penicillin) at 37 °C in a 5% CO2 environment. All human trophoblast cell lines had been authenticated using STR profiling within the previous three years. All experiments were performed with mycoplasma-free cells.

PKM2-K305R and PKM2-WT plasmid were purchased from GeneChem (GeneChem, Shanghai, China). Jar cells were transfected with over-expression plasmid or control plasmid using Lipo2000 transfection reagent (Invitrogen, USA) according to the manufacturer’s instructions. The amount of plasmid transfection in a single well (6-well plate) was 2.5 µg. After 48 h, the cells were harvested and used for subsequent experiments.

### Over-expression lentivirus infection

HLA-F over-expression lentivirus (NM_018950), prepared using the vector element sequence Ubi-MCS-3Flag-CBh-gcGFP-IRES-Puromycin, was purchased from GeneChem (GeneChem, Shanghai, China). The lentivirus was transduced into cells according to the manufacturer’s instructions. Virus-containing media was replaced with fresh culture medium in wells after 24 h of infection. DMEM culture medium containing 10% FBS and 2 mg/ml puromycin was added to culture the infected cells for another 24 h.

### siRNA, shRNA transfection

Jar cells or HLA-F-overexpression Jar cells were transfected with either shRNA and siRNA that targeted HLA-F and PKM2 or a nonspecific control siRNA sequence using ribo-FECTTMCP Reagent (Guangzhou Ribobio Co., Ltd, Guangzhou, China) according to the manufacturer’s protocol and analyzed three days later. The HLA-F shRNA target gene sequences were as follows: #1: CGCAGTAT TGGGAGTGGACCACTCGAGTGGTCCACTCCCAATACTGCGTTTTTG, #2: CCGGAGAGGAATATGCAGAGGAGTTCTCGAGAACTCCTCTGCATATTCCTCTTTTTTG, #3: CCGCCGGAGATGCGTAATGTGGAAACTTCAAGAGAAGTTCC.

ACATTACGCATCTCCTTTTTG. The PKM2 siRNA target gene sequences were as follows: #1, 5′-CCAACACCATGCGTGTTGT-3′, #2, 5′ GGATGTTGATATGGTGTTT-3′, and #3, 5′-GTGGTGATCTAGGCATTGA-3′.

### Colony formation assay

The colony formation assay was performed as previously described (Braselmann et al. 2015). Briefly, 200 infected cells were cultured in six-well plates at 37 °C for 7–10 days. Visible colonies were washed twice with PBS, fixed with 4% paraformaldehyde, and stained with crystal violet. Images of the colonies were taken, and the number of colonies was counted by ImageJ software.

### MTT

The proliferation of cells was assessed by 3-(4,5-dimethylthiazol‐2‐yl)‐2,5‐diphenyltetrazolium bromide (MTT) assay. Briefly, cells were transfected with either control shRNA or shRNA against HLA-F. After 24 h, cell viability was measured by the MTT assay.

### Co-IP

Immunoprecipitation (IP) was performed according to the manufacturer’s instructions for the Pierce™ Classic Magnetic IP/Co-IP Kit (Thermo Fisher, 88804). First, the corresponding primary antibody was incubated with cell lysate overnight at 4 °C to form the immune complex. Then, Pierce Protein A/G Magnetic Beads were incubated with the antigen sample and antibody mixture at room temperature for 1 h with mixing. Finally, the bound protein was eluted for western blot analysis. The primary anti-Lactyllysine (PTM-BIO, #PTM-1401RM) was utilized to assess the levels of pan-lactyllysine (Pan-Kla) in Jar cells. Additionally, an anti-PKM2 primary antibody (Proteintech, #60268-1-Ig) was employed for further analysis.

### CHIP-seq

The Chromatin Immunoprecipitation and deep sequencing (ChIP-seq) assay was performed according to the manufacturer’s instructions of SimpleChIP Enzymatic Chromatin IP kit (CST, Danvers, MA, USA) The chromatin of treated cells was fixed, lysed and sonicated. The end repair of DNA after IP is performed by adding base A. The repaired, A-added DNA was treated with sequencing splices. The target size fragments were recovered by magnetic beads and amplified by PCR to obtain the library to be sequenced. The constructed library was inspected by agarose electrophoresis. Qubit 2.0 was used to quantify the library and determine whether the concentration of the library was suitable for the computer. After the DNA library was qualified, the different libraries were sequenced on Illumina Hiseq 3000 sequencer according to the requirements of effective concentration and target data volume. Anti-Flag antibody (Bioss, bsm-33346 M, Chian) and IgG (CST, Danvers, MA, USA) were used to enrich HLA-F-binding DNA fragments. The HLA-F-binding PKM2 promoter region was verified by qPCR and AGAR electrophoresis.

### 4D-LFQP-LA

The 4-dimensional label-free quantitative proteomics combined with lactylation analysis (4D-LFQP-LA) was supported by Jingjie PTM BioLabs (Hangzhou, China). In brief, Control-OE and HLA-F-OE Jar cells were re-suspended and lysed. The tryptic peptides were dissolved in solvent A (0.1% formic acid, 2% acetonitrile/in water) and separated by a homemade reversed-phase analytical column (25 cm length, 100 μm i.d.) on a nanoElute UHPLC system (Bruker Daltonics). After being subjected to a capillary source, the peptides were subjected to timsTOF Pro (Bruker Daltonics) mass spectrometry for further analysis in parallel accumulation serial fragmentation (PASEF) mode. The MS/MS data were processed by the Max Quant search engine (v.1.6.6.0). Tandem mass spectra were searched using the human SwissProt database (20,366 entries) and reverse decoy database. FDR < 1%. The relative quantitative values of modified peptides in different samples were obtained by centralizing the signal intensity values in different samples. After filtering lysine lactylation sites (localization probability > 0.75), the relative quantitative values of each sample were obtained by two experiments. All the ratios of quantified lysine lactylation peptides were normalized according to their corresponding protein expression levels. The protein pathways were annotated using the KEGG database.

### MiniPDX

Mini patient-derived xenograft (Mini-PDX) assay was performed using the OncoVee^®^-MiniPDX kit (LIDE Biotech Co., Ltd, Shanghai, China). Five- to six-weeks-old immunodeficient female BALB/c-Nude mice were purchased from SLARC Inc., Shanghai. All animal experiments were approved by the Institutional Animal Care and Use Committee of Sichuan Provincial People’s Hospital University. Sh-HLA-Jar cells were enriched and filled into OncoVee^®^ MiniPDX capsules (LIDE Biotech Co., Ltd, Shanghai, China). Approximately 2000 cells are contained in each capsule. By making a small incision on the skin of a mouse, the capsules are implanted subcutaneously. Three capsules are implanted in each mouse. There are six capsules for each group of drugs and a total of two mice. Mice bearing OncoVee^®^ MiniPDX capsules are administered PKM2 agonist (50 mg/kg, intraperitoneal injection on days 1, 3, and 5) or the same dose of saline as control. After seven days of drug treatment, all capsules are taken out from the mice. The proliferation condition of cells in each capsule is measured using the CellTiter Glo Luminescent Cell Viability Assay kit (G7571, a product of Promega in Madison, Wisconsin, USA). The relative proliferation T/C- ratio was calculated in reference to the previous research (Ge et al. 2022; Cui et al. 2024).

### Indirect enzyme-linked immunosorbent assay (ELISA)

The concentration of pyruvate and lactate of intracellular and extracellular Jar cells and the placenta tissue were tested using a pyruvate (PA) content assay kit and a Lactic acid (LD) Content assay kit (Beijing Solarbio Science & Technology Co., Ltd, BC2205&BC2230) following the manufacturer’s instructions. In brief, the Jar cells and their medium (supernatant) were harvested and used for measuring the concentration of PA and LD, which was calculated according to standard curves created using standard samples provided by the kit manufacturer.

### PK activity assay

For the intracellular PK activity assay, Jar cells were re-suspended and lysed. The supernatant was then used in the enzyme assays using a Pyruvate Kinase (PK) Activity Assay Kit (Beijing Solarbio Science & Technology Co., Ltd, BC0540), following the manufacturer’s instructions. Pyruvate kinase activity was normalized to protein concentration. For the extracellular PK activity assay, the supernatant of Jar cell was used in the enzyme assays directly.

### Immunofluorescence staining and quantification

A cover glass was placed in the bottom of the 6-well plate. After 72 h, the adherent Jar cells on the cover glass were fixed with 4% paraformaldehyde for 30 min. Immunostaining blocking solution was dropped on at room temperature for 10–20 min and then removed, followed by incubation with rabbit monoclonal to PKM (Abcam, EPR10138(B), 1:50) and rabbit anti-HLA-F antibody monoclonal antibody (Bioss, bs-17544R, 1:100) respectively at room temperature for 1 h or overnight at 4℃ and then anti-mouse 488 fluorescent secondary antibody at room temperature for 1 h. Finally, 1 ml nuclear staining (DAPI) was added to stain at room temperature for 5 min. Image J was used to analyze the mean fluorescence intensity of HLA-F and PKM2. The distribution of cytoplasmic and nuclear fluorescence of PKM2 in Jar cells was visualized by the radial (α to γ) grayscale measurement function of Image J software.

### Statistical analysis

All data were expressed as means ± SD prior to being analyzed by two-tailed paired Student’s t-test unless otherwise indicated. The threshold for statistically significant results was set to *p* < 0.05. Sample sizes of placenta tissues were evaluated according to the power analysis under the prior effect size, and p-value threshold with R package pwr (version 1.3.0). All statistical analysis was carried out in GraphPad Prism 9 software (GraphPad Software Inc. USA).

## Results

### Expression of HLA-F is reduced in trophoblast from patients with preeclampsia

**Fig. 1:**
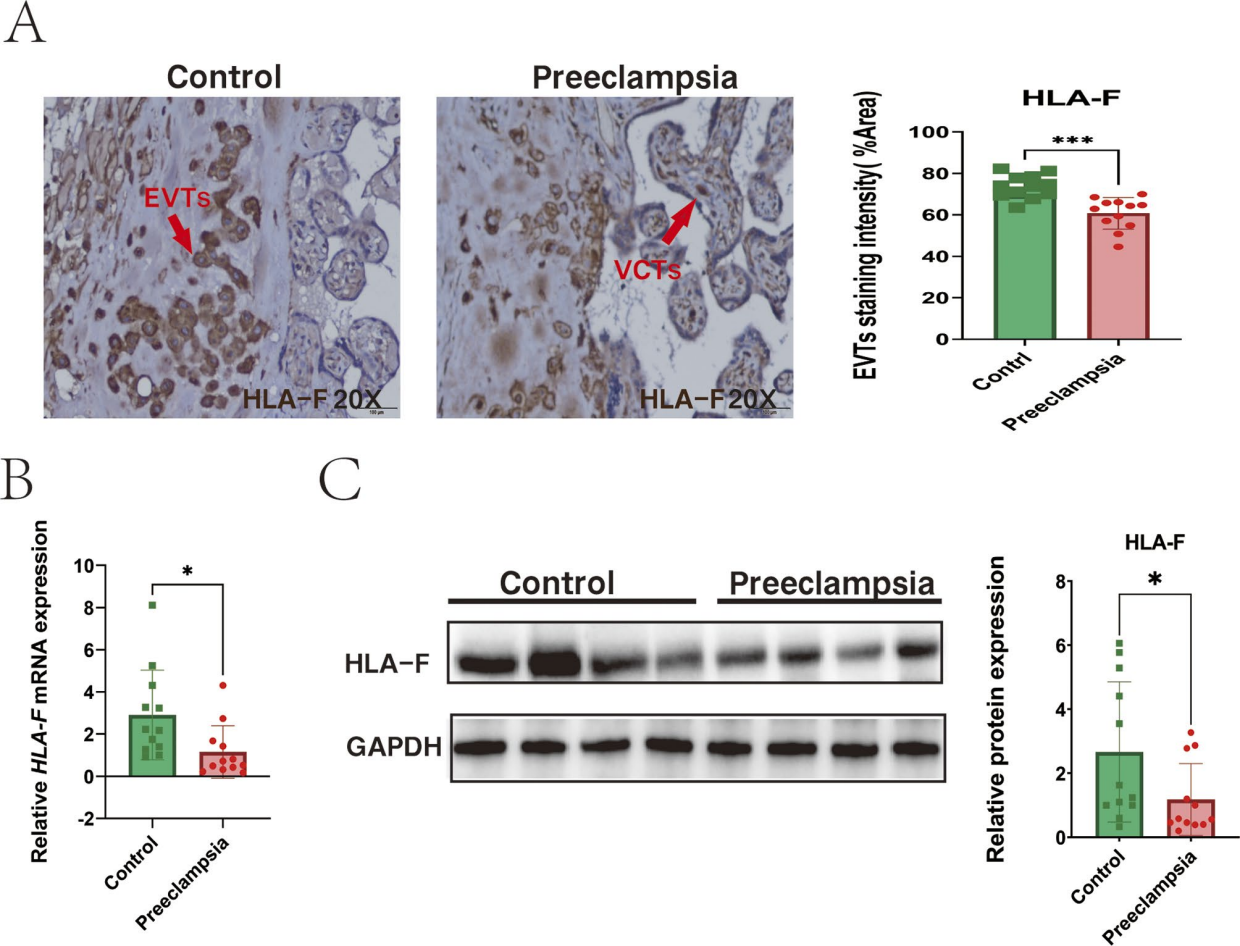
The expression of HLA-F was lower in trophoblast cells from the placenta of patients with preeclampsia. (**A**) HLA-F immunostaining of EVT of the placenta bed from tissues taken from patients with preeclampsia (*n* = 12) and normal term controls (*n* = 12). Three EVT cells were randomly selected by investigators blinded to the patient group, and Image-J software was used to measure the %Area value of the cells. The mean value of %Area of 3 EVT cells was used to represent the staining intensity of HLA-F expression in the sample EVT. Statistical analysis showed that HLA-F expression was lower in EVT in the preeclampsia group compared to controls. (**B**) qPCR confirmed that the relative mRNA expression of HLA-F in the placenta of preeclampsia patients (*n* = 12) was significantly lower than in normal term controls (*n* = 12). (**C**) Western blot analysis demonstrated that the relative protein expression of HLA-F in the placenta of preeclampsia patients (*n* = 12) was significantly lower than in normal term control (*n* = 12). Asterisks indicate significant differences (ns: no significance, *p < 0.05, **p < 0.01, ***p < 0.001)

To confirm previously reported results regarding HLA-F expression at the maternal-fetal interface, we performed HLA-F immunohistochemical staining on samples of placenta obtained from 12 patients with preeclampsia and 12 normal late-pregnancy controls. The clinical data can be found in Table S2. This staining demonstrated that EVT cells in decidua and the villous cytotrophoblast (VCT) were HLA-F positive, with HLA-F staining observed in the plasma membrane, cytoplasm, and some nuclei. EVT cells from preeclamptic samples displayed a lower intensity of HLA-F staining compared with that observed in normal nonlaboring term EVTs (Fig. 1A). Next, we used PCR and Western blot to quantify HLA-F expression in VCT cells obtained from the placenta of preeclampsia patients and normal late pregnancy controls. These analyses confirmed that the expression of HLA-F mRNA and protein was significantly lower in the placenta of preeclampsia patients than that of late pregnancy controls (Fig. 1B-C).

### HLA-F promotes trophoblast cell proliferation

**Fig. 2:**
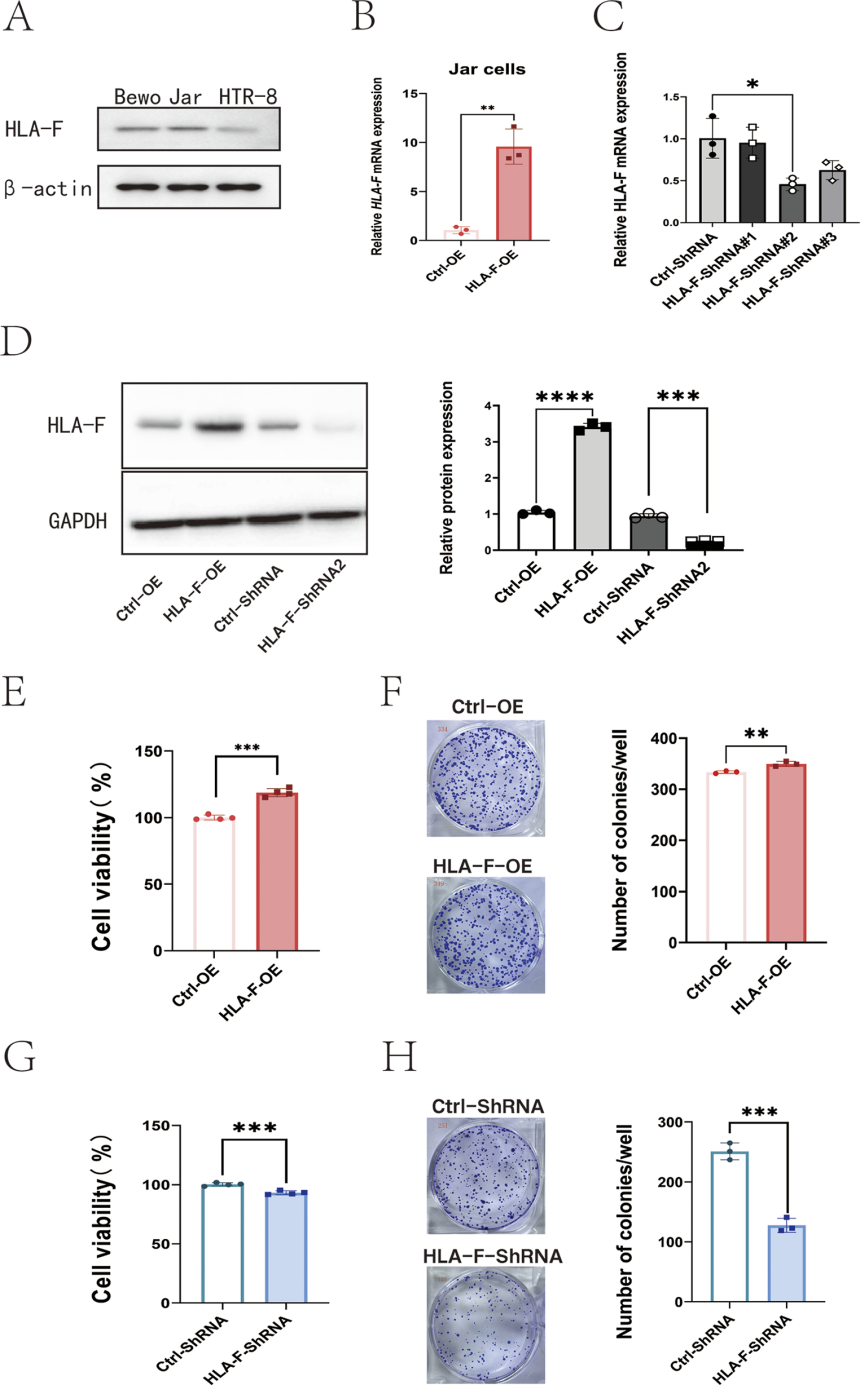
HLA-F promotes Jar cell proliferation. (**A**) Western blot showing the protein expression of HLA-F in BeWo, Jar, and Htr8/SVneo cell lines. (**B**) qPCR confirmed that the relative expression of HLA-F mRNA was significantly higher in Jar cells transfected with an HLA-F–overexpressing lentivirus (HLA-F-OE) compared to cells transfected with the control lentivirus (Ctrl-OE). (**C**) Western blot demonstrated that the relative protein expression was also significantly up-regulated in the HLA-F-OE group. (**D**) MTT assay indicated that the viability of Jar cells in the HLA-F-OE group was significantly higher than in the Ctrl-OE group. (**E**) Colony formation assay revealed that the number of colonies was significantly higher in the HLA-F-OE group than in the Ctrl-OE group. (**F**) Jar cells were transfected with either ctrl-siRNA or HLA-F-siRNA, and the relative expression of HLA-F mRNA was assessed by qPCR. Transfection with the #2 HLA-F siRNA target gene sequence significantly decreased expression of HLA-F mRNA. (**G**) Western blot confirmed that HLA-F protein expression was significantly suppressed in Jar cells transfected with the #2 HLA-F siRNA target gene sequence. (**H**) MTT assay demonstrated that the viability of Jar cells in the siRNA-HLA-F group was significantly lower than in the siRNA-Ctrl group. (**I**) Colony formation assay showed that the number of colonies was significantly lower in the siRNA-HLA-F group than in the siRNA-Ctrl group. Three replicates were used for each experiment. Data are expressed as mean ± SD. A line connects the pairs of groups that were significantly different from each other, and T-tests were used for comparison between the two groups. Asterisks indicate significant differences (ns: no significance, *p < 0.05, **p < 0.01, ***p < 0.001)

Using Western blot, we examined the levels of HLA-F in different trophoblast cell lines, including Bewo and Jar cells, which are derived from human placental villus carcinomas, and HTR-8/SVneo cells, which are derived from EVTs. We observed that HLA-F expression was higher in Bewo and Jar cells than in HTR-8/SVneo cells (Fig. 2A), which confirmed previous reports, based on scRNA-seq, that HLA-F expression was higher in VCT than in EVT (Luo et al. 2023). Our preliminary studies using the CCK-8 assay demonstrated that transfection with HLA-F overexpression plasmids enhanced the proliferative capacity of Jar cells, whereas transfection with HLA-F siRNA reduced cell proliferation (Luo et al. 2023). To further validate these findings, we employed an HLA-F overexpression lentivirus and an HLA-F-targeting shRNA to upregulate and downregulate HLA-F expression in Jar cells, respectively. The results confirmed significant alterations at both mRNA and protein levels (Fig. 2B-D). The viability and colony numbers of HLA-F–overexpressing Jar cells (HLA-F-OE) were significantly superior to those of control vector-overexpressing Jar cells (Ctrl-OE) (Fig. 2E-F). After Jar cells were transfected with ShRNA targeting HLA-F (HLA-F-ShRNA), as expected, the proliferation of those trophoblast cells was significantly suppressed (Fig. 2G-H).

### HLA-F promotes PKM2-mediated glycolysis of trophoblast cells

**Fig. 3:**
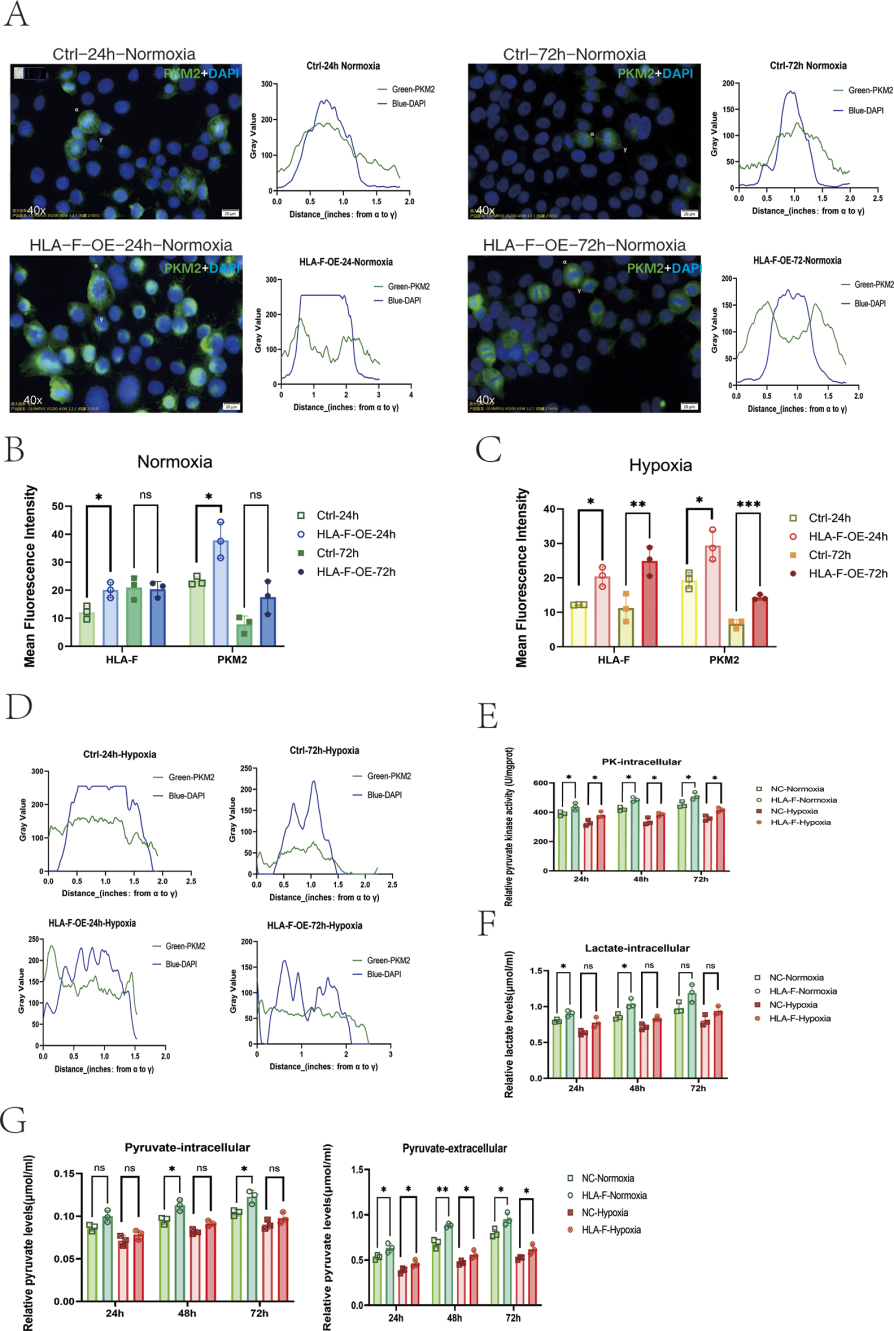
Upregulated expression of HLA-F results in enhanced glycolysis and PKM2 enzyme activity in Jar cells. (**A**) Immunofluorescence staining revealed the cytoplasmic and nuclear distribution of PKM2 in HLA-F–overexpressing Jar cells (HLA-F-OE group) and control Jar cells (Ctrl group) under normal oxygen culture. The nuclei were stained with DAPI (blue) and the PKM2 was labeled and visualized using a green fluorescent marker. The line charts represent fluorescence intensity (gray value), corresponding to the green signal (PKM2) and the blue signal (DAPI) were measured and plotted along the designated distance from α to γ. (**B**) Analysis of mean fluorescence intensity (MFI) of HLA-F and PKM2 protein expression in Jar cells from the HLA-F-OE group and Ctrl group under normoxic conditions at 24 and 72 h. After 24 h of normal oxygen culture, the expression of HLA-F and PKM2 proteins was significantly higher in the HLA-F-OE group than in the Ctrl group. **p < 0.05. (**C**) MFI of HLA-F and PKM2 protein expression in the two groups of Jar cells at 24 and 72 h under hypoxic conditions; HLA-F and PKM2 protein expression was significantly higher in the HLA-F-OE group than in the Ctrl group after 24 and 72 h of hypoxic culture. (**D**) A line chart depicting the cytoplasmic and nuclear distribution of PKM2 in the HLA-F-OE group and Ctrl group under hypoxic culture. The line charts represent fluorescence intensity (gray value), presenting the distance from α to γ. (**E**) A PK enzyme activity assay kit was used to assess the intracellular PKM enzyme activity of HLA-F-OE and Ctrl (NC) Jar cells after 24 h, 48 h, and 72 h of normoxic and hypoxic culture. Intracellular PKM enzyme activity was significantly higher in the HLA-F-OE group than in the Ctrl (NC) group. (**F-G**) Intracellular and extracellular content of lactic acid and pyruvate in HLA-F-OE and NC Jar cells as detected by ELISA after 24 h, 48 h, and 72 h of culture under normoxic and hypoxic conditions. Three replicates were used for each experiment. Data are expressed as mean ± SD. A line connects pairs of groups that were significantly different from each other, and T-tests were used for comparisons between the two groups. Asterisks indicate significant differences (ns: no significance, *p < 0.05, **p < 0.01, ***p < 0.001)

In protein spectrometry analyses of HLA-F–overexpressing Jar cells, we detected a significant increase in the expression of PKM2, a key enzyme of the glycolysis pathway, along with the PI3K/AKT pathway–related proteins PIK3R1, PIK3CB, AKT1, and AKT2 (Table S3). As the process of glycolysis, especially aerobic glycolysis, provides the initial energy supply for early embryo implantation and growth (Bloxam et al. 1987), we next investigated whether PKM2-mediated changes in glycolysis were associated with the increased proliferation of HLA-F–overexpressing Jar cells. For this, we cultured HLA-F–OE Jar cells and control Jar cells for 72 h under either normal oxygen or hypoxic (2% O_2_) conditions. Differences in cellular localization and expression intensity of HLA-F and PKM2 proteins were detected by immunofluorescence staining, and the activity of PKM enzymes (PK), lactic acid (LD), and pyruvate (PA) was detected by ELISA. In HLA-F-OE Jar cells cultured under normoxic conditions, quantitative analysis of immunofluorescence staining revealed the increased presence of PKM2 in cytoplasm after 24 and 72 h, likely representing an increase in enzymatic activity (Fig. 3A). The average fluorescence intensity of HLA-F and PKM2 proteins was significantly higher in HLA-F-OE Jar cells compared to controls after 24 h of normoxic culture or after 72 h of hypoxic culture (Fig. 3B-C). The shift in distribution of PKM2 from the nucleus to the cytoplasm of HLA-F-OE Jar cells also tended to be more marked under hypoxic conditions, but it was not obvious at 72 h (Fig. 3D). Immunofluorescence data were supported by the results of ELISA assays, which confirmed that PK levels were higher in HLA-F-OE Jar cells than in control Jar cells in both hypoxic and normoxic conditions (Fig. 3E). After 24 and 48 h of normoxic culture, the lactate content of HLA-F-OE Jar cells was significantly higher than in control Jar cells (Fig. 3F). With respect to pyruvate, higher levels were detected in HLA-F-OE Jar cells than in control Jar cells only after 48 and 72 h of normal oxygen culture, but the pyruvate content of extracellular exudates of HLA-F-OE Jar cells was significantly higher than that of control Jar cells under both normoxic and hypoxic conditions (Fig. 3G). These observations suggested that HLA-F might play an important role in PKM2-mediated glycolysis.

### HLA-F induces PKM2 expression

**Fig. 4:**
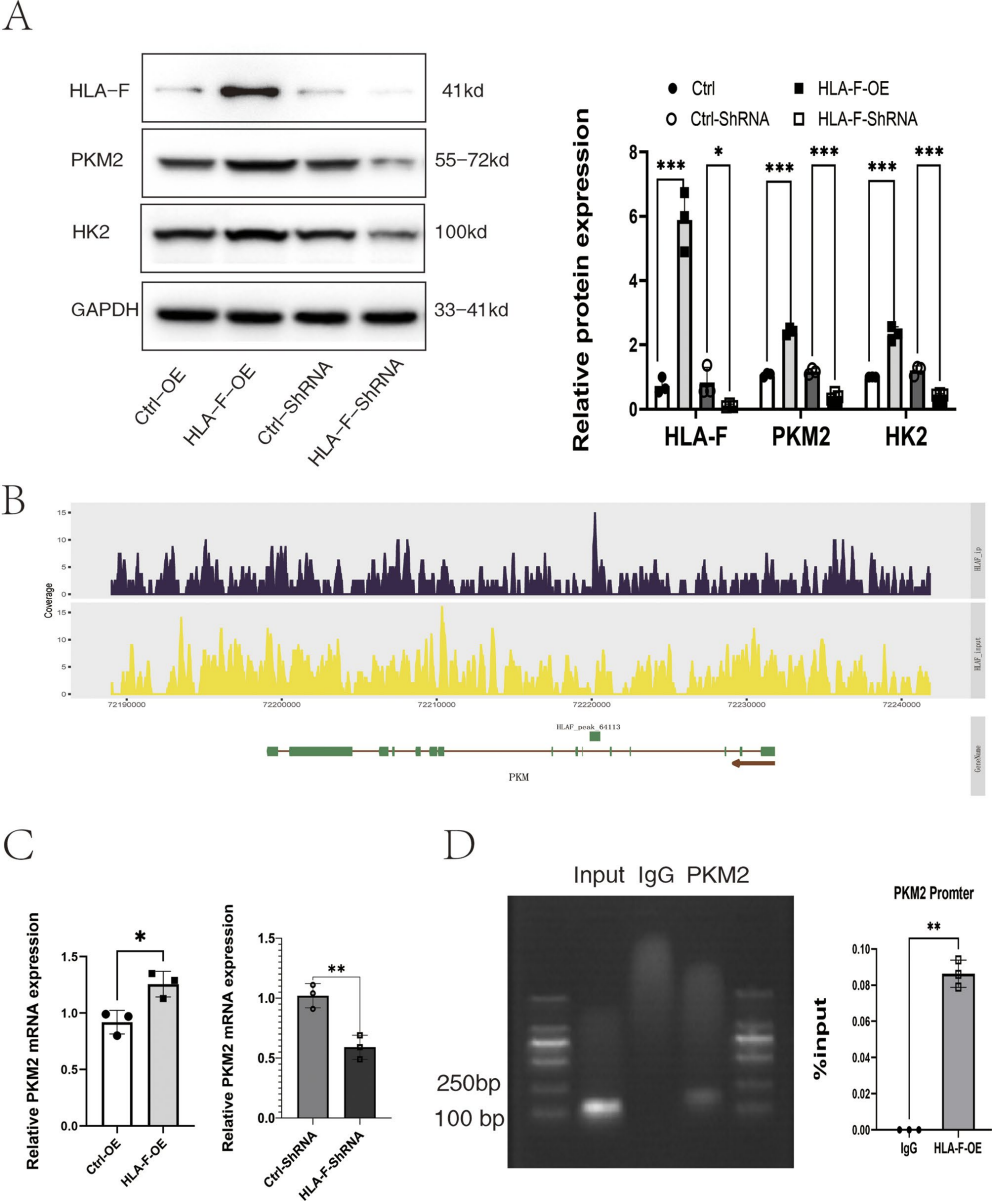
HLA-F induces PKM2 protein expression in Jar cells. (**A**) Western blot was used to detect the expression of PKM2 and HK2 proteins in Jar cells transfected with an HLA-F–overexpression lentivirus and/or HLA-F–interferon siRNA. PKM2 and HK2 protein expression were both significantly higher in the HLA-F-OE group than in the Ctrl-OE group, and both were significantly lower in the HLA-F–siRNA group than in the Ctrl-siRNA group. (**B**) In the ChIP-seq analysis of transcription factors with Input and IP, we used Input as the background and Macs2 for IP peak calling in narrow peak mode. The peak chart shows HLA-F_ peak reads in the PKM promoter region. (**C**) qPCR verified that PKM mRNA expression was significantly higher in Jar cells that overexpressed HLA-F and significantly lower in Jar cells in which HLA-F expression was suppressed. (**D**) Electrophoretic images of qPCR products generated from the chromatin precipitation from ChIP analyses (left). The %input value was calculated based on the CT values of IP and IgG and the input dilution ratio (right). Three replicates were used for each experiment. Data are expressed as mean ± SD. A line connects the pairs of groups that were significantly different from each other, and T-tests were used for comparison between the two groups. Asterisks indicate significant differences (ns: no significance, *p < 0.05, **p < 0.01, ***p < 0.001)

In order to investigate the mechanism by which HLA-F might regulate PKM-mediated glycolysis, we first verified the protein expression of the key glycolytic enzymes PKM2 and HK2 in Jar cells in which HLA-F was either overexpressed or inhibited. Western blot revealed that levels of PKM2 and HK2 were significantly higher in HLA-F–overexpressing Jar cells compared with control-overexpressing Jar cells, while, on the other hand, targeting HLA-F with shRNA suppressed PKM2 and HK2 protein expression compared to cells transfected with control shRNA (Fig. 4A). To examine this further, we performed ChIP-seq using HLA-F-OE Jar cells (Table S4). These results demonstrated the presence of an HLA-F binding peak (HLA-F_peak_64113) in the promoter region of *PKM*, indicating that HLA-F might promote the transcription of PKM2 by binding to the PKM gene promoter (Fig. 4B). To verify the results of ChIP-seq, real-time fluorescence quantitative PCR was performed on Jar cells with HLA-F overexpression or inhibition. We found that *PKM* mRNA expression was significantly higher in HLA-F–overexpressing Jar cells and significantly lower in HLA-F–inhibited Jar cells (Fig. 4C). Finally, we used qPCR to search for the *PKM* promoter region in the chromatin precipitation from ChIP, and found that it was indeed present (Fig. 4D); this confirmed that the HLA-F protein promotes the transcription of PKM by binding to its promoter region.

### HLA-F promotes proliferation by regulating PKM2

**Fig. 5:**
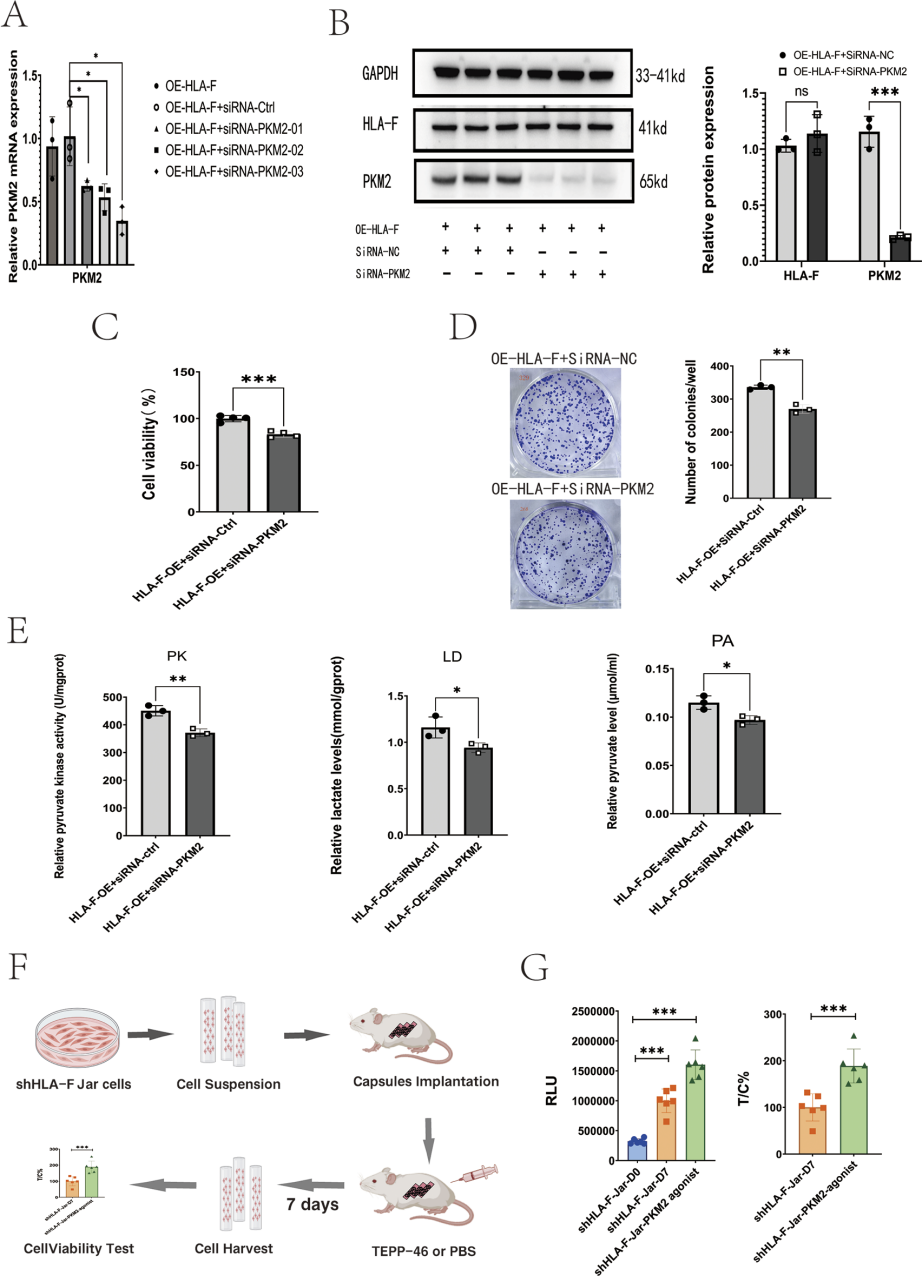
PKM2 is required for HLA-F–mediated proliferation and glycolysis. (**A**) Expression of PKM mRNA in HLA-F-OE Jar cells transfected with either PKM2 siRNA or ctrl siRNA, as detected by PCR. siRNA sequences 1–3 significantly decreased PKM mRNA expression. (**B**) Western blot revealed that PKM2 protein expression was significantly lower in HLA-F-OE Jar cells transfected with PKM2 siRNA, while HLA-F protein expression remained unchanged. (**C**) CCK8 assay indicated that the cell viability of HLA-F-OE Jar cells transfected with PKM2 siRNA was significantly lower than that of cells transfected with control siRNA. (**D**) Colony formation assay showed that the number of colonies of HLA-F-OE Jar cells was significantly lower when treated with siRNA-PKM2 than with siRNA-Ctrl. (**E**) PKM enzyme activity and the content of lactic acid and pyruvate were significantly reduced in HLA-F-OE Jar cells transfected with PKM2 siRNA than in cells transfected with control siRNA. Three replicates were used for each experiment. Data are expressed as mean ± SD. A line connects the pairs of groups that were significantly different from each other, and T-tests were used for comparison between the two groups. Asterisks indicate significant differences (ns: no significance, *p < 0.05, **p < 0.01, ***p < 0.001)

To verify that the effect of HLA-F on trophoblast proliferation is mediated through the expression and enzymatic activity of PKM2, we used siRNA to silence PKM2 in HLA-F-OE Jar cells and then performed ELISA and proliferation assays. Each of the three kinds of PKM2-targeting siRNA we tested was able to reduce PKM2 expression in HLA-F-OE Jar cells (Fig. 5A); for subsequent experiments we utilized PKM2-siRNA03. Western blot confirmed a significant decrease in the expression of the PKM2 protein (Fig. 5B). As expected, the knockdown of PKM2 effectively decreased the viability and colony number of HLA-F-OE Jar cells compared with cells transfected with control siRNA (Fig. 5C-D). Additionally, ELISA results revealed that the downregulation of PKM2 expression in HLA-F-OE Jar cells significantly suppressed lactate and pyruvate production and decreased the activity of PKM2 (Fig. 5E). Finally, we used a MiniPDX model to verify the effect of a PKM2 agonist, TEPP-46, on the proliferation of HLA-F–knockdown Jar cells. The experimental workflow of the MiniPDX assay is illustrated in Fig. 5F. Consistent with the results described above, the PKM2 agonist was able to increase the cell viability of HLA-F–knockdown Jar cells in vivo (Fig. 5G).

### HLA-F promotes PKM2 enzyme activity by inhibiting PKM2 K305 lactylation

**Fig. 6:**
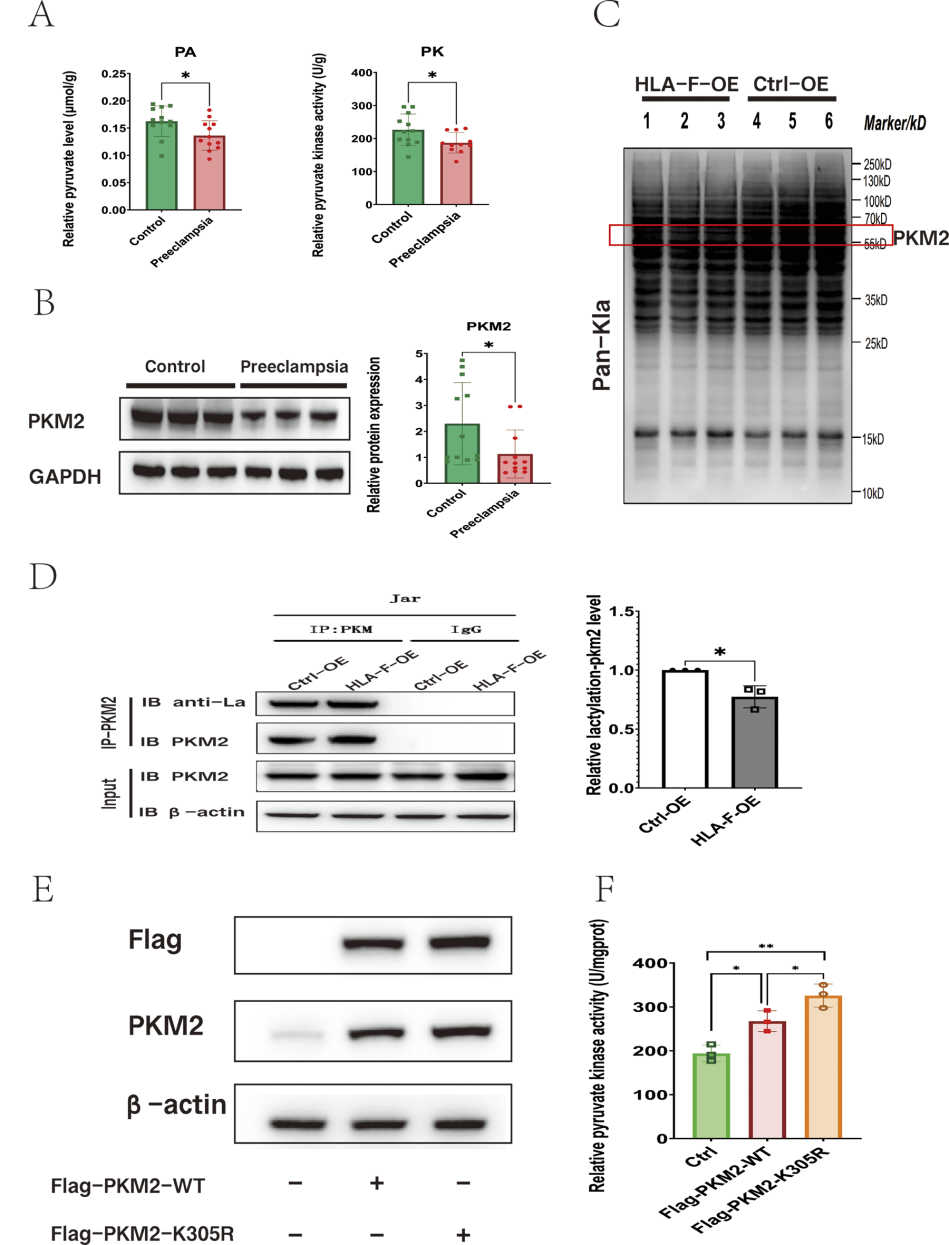
HLA-F is involved in suppressing the lactylation of PKM2 K305. (**A**) PKM enzyme activity and pyruvate content were significantly lower in placental tissues taken from patients with preeclampsia (*n* = 12) than in those from normal term controls (*n* = 12). (**B**) Results of Western blot illustrating that the relative protein expression of PKM2 was significantly reduced in placenta from patients with preeclampsia (*n* = 12) compared to that of normal term controls (*n* = 12). (**C**) Western blot results of the lactylation level of proteins extracted from HLA-F–overexpressing (HLA-F-OE) Jar cells or control-overexpression (Ctrl-OE) Jar cells using anti-lactyllysine (pan-kla). The red box contains the PKM2 protein. (**D**) The proteins from HLA-F-OE Jar cells or Ctrl-OE Jar cells were bound by a PKM2 antibody and lactylation was detected using an anti-lactyllysine antibody. The relative lactylation-PKM2 level was calculated based on an OD value of IP-PKM2-PKM2/IP-PKM2-anti-la. The Co-IP experiment was replicated three times. (**E**) Western blot of Jar cells transfected with a plasmid that overexpressed either PKM2-WT or PKM2-K305R. (**F**) PKM enzyme activity assay of Jar cells transfected with control, PKM2-WT–, or PKM2-K305R–overexpressing plasmid. Three replicates were used for each experiment. Data are expressed as mean ± SD. A line connects the pairs of groups that were significantly different from each other, and T-tests were used for comparison between the two groups. Asterisks indicate significant differences (ns: no significance, *p < 0.05, **p < 0.01, ***p < 0.001)

To evaluate the hypothesis that insufficient HLA-F expression results in the reduced proliferation of trophoblastic cells via the down-regulation of PKM2-mediated glycolysis, we first assessed PKM2 enzyme activity, pyruvate content, and PKM2 protein expression in placental tissues obtained from patients with preeclampsia (*n* = 12) and normal late pregnancy controls (*n* = 12). Consistent with the results of our in vitro experiments, PKM2 activity, and pyruvate content were significantly lower in the placenta of preeclampsia patients than in that of normal controls (Fig. 6A), and PKM2 protein expression was also significantly decreased (Fig. 6B). We then performed another set of experiments on engineered Jar cells to explore the specific mechanism by which HLA-F can regulate PKM2 activity. As HLA-F can promote glycolysis and lactate generation, and lactate can regulate the enzymatic activity of PKM2 through the lactylation of lysine (Wang et al. 2022), it may be that HLA-F affects the lactic modification of PKM2. To investigate this, we examined Pan-Kla levels in HLA-F-OE Jar cells (HLA-F-OE group) and control-overexpressing Jar cells (Ctrl-OE group). We observed differences in lactylation in proteins of various sizes, some of which were consistent with the size of the PKM2 protein, i.e., about 65 kd (Fig. 6C). To describe the lactylation profiles of the different proteins in the two groups of cells, we employed 4D-LFQP-LA; the results confirmed the down-regulation of PKM2 lactylation at lysine residue 305 (PKM2 K305lac) in the HLA-F-OE group (Table S5). Next, we used the PKM2 antibody to bind PKM2 and used the anti-lactyllysine antibody to detect the level of PKM2 lactylation. Consistent with the 4D-LFQP-LA results, we observed that the level of lactylation on PKM2 was significantly lower in HLA-F–overexpressing Jar cells (Fig. 6D). Since PKM2 activity was significantly higher in the HLA-F-OE group, we speculated that K305lac inhibited its enzymatic activity. To test this hypothesis, we constructed flag-tagged plasmids that overexpressed PKM2 K305R or PKM2 WT and transfected them into control Jar cells. As shown in Fig. 6E, compared with the control group, PKM2-overexpressing cells exhibited significantly up-regulated expression of PKM2 protein. Furthermore, Jar cells with the K305R mutation demonstrated significantly higher PKM2 enzyme activity (Fig. 6F). Together, these results demonstrated that the K305 site has an important effect on the function of PKM2 and that HLA-F promotes the activity of PKM2 by inhibiting the lactylation of this site.

## Discussion

Of all the HLA genes, the nonclassical HLA-Ib molecules—including HLA-F, HLA-E, and HLA-G—are the most conserved. These molecules play a role in immune evasion strategies and/or act as mediators of immune tolerance, and are therefore characterized by their restricted tissue distribution (Hò et al. 2019); they also play a key role in antivirus and anti-infection responses through their classical antigen-presenting function (Garcia-Beltran et al. 2016). It has been reported that the expression levels of HLA-F, HLA-E, and HLA-G are all regulated by gene polymorphisms (Zidi et al. 2016), such as SNP rs2523393 of HLA-F, for which the AA genotype exhibits a higher expression of both HLA-F and HLA-G (Mika et al. 2018). Individuals carrying the F*distal-C haplotype in the *HLA-F* promoter exhibit elevated *HLA-F* mRNA expression levels, and this haplotype is linked to the F*01:01:02 alleles (Buttura et al. 2019). Higher HLA-F expression in the decidua has been associated with improved pregnancy success rates and reduced time-to-pregnancy following fertility treatments (Hviid et al. 2020; Papúchová et al. 2022). In addition to gene polymorphism, previous studies have also found that HLA-F, G, and E have their own specific transcription factors. For example, HLA-F expression is regulated by NF-κB and IFN-γ, while HLA-E is regulated by IFN-γ and STAT1 (Elsen 2000; Ismael et al. 2024). Dunk et al. observed decreased HLA-F expression in EVTs from patients with preeclampsia, whereas the highest levels of HLA-F expression were detected in patients with chorioamnionitis (Dunk et al. 2022). These findings align with the regulation of HLA-F expression by NF-κB and IFN-γ. In the context of bacterial or viral infections, elevated levels of IFN-γ and NF-κB upregulate HLA-F expression, facilitating immune regulation. In this context, it seems likely that the decreased expression of HLA-F in the trophoblast of preeclampsia patients may be the result of gene polymorphism. It is plausible that genotypes associated with elevated HLA-F expression levels, such as the F*01:01:02 alleles and the SNP rs2523393 AA genotype, may be less prevalent among patients with preeclampsia. However, the potential association between HLA-F gene polymorphisms and preeclampsia remains unexplored and warrants further investigation.

Although the precise role of HLA-F in reproduction and during pregnancy has yet to be systematically studied (Persson et al. 2020), recent research has shed light on the relationship between this molecule and tumor progression. Studies have found that HLA-F-AS1 promotes colorectal cancer progression via regulation of the miR-330-3p/PFN1 axis (Huang et al. 2020) and that expression levels of HLA-F are negatively correlated with the prognosis of brain glioma (Feng et al. 2019). More specifically, it was reported that HLA-F promotes glioma cell proliferation via HK2-dependent glycolysis (Chen et al. 2021a). This research may have applications to the study of pregnancy since embryonic trophoblast cells share certain characteristics with tumor cells. Our previous study found that HLA-F can promote the proliferation of Jar cells (Luo et al. 2023). In the present study, we confirmed the effect of HLA-F on the proliferation of Jar cells by employing MTT, clonal formation, and MiniPDX. Trophoblast proliferation is closely related to placentation, and many studies have found that abnormal trophoblast proliferation can cause preeclampsia (Liu et al. 2023; Cui et al. 2020; Xu et al. 2018), fetal growth restriction (Zhang et al. 2023; Li et al. 2018), and recurrent spontaneous abortion (Zhu et al. 2022; Xiao et al. 2022). Studies of the relevant molecular mechanisms have focused on glycolysis/PI3K/AKT (Zhu et al. 2022), MAPK (Liu et al. 2023), and TGF-β(Xiao et al. 2022), among others.

The process of glycolysis provides the primary energy for the implanted embryo, and expression of the glycolysis-associated genes HK, PKM, and LDHA was found to be significantly higher at the implantation site than in other parts of the decidua (Zuo et al. 2015). In HTR-8/SVneo trophoblast cells, deficiencies in lactate dehydrogenase (LDHA) were reported to lead to impaired glycolysis and the down-regulation of the PI3K/AKT pathway, thus reducing their proliferation ability (Zhu et al. 2022). Abnormalities in glycolysis have also been associated with the onset of preeclampsia. For example, placental tissues from preeclampsia patients were reported to contain significantly reduced levels of pyruvate and lactate (Bloxam et al. 1987). A recent study linked preeclampsia with decreased expression of the RNA-binding protein SORBS2, which promotes glycolysis, cell proliferation, and migration of HTR-8/SVneo cells by increasing the stability of the HK2 enzyme (Song L 2024). Taken together, these studies all suggest a role for abnormal trophoblast glycolysis in the pathogenesis of preeclampsia. In the present study, we observed that HLA-F can promote the protein expression and enzyme activity of PKM2, upregulate the glycolysis pathway, and promote the proliferation of trophoblast cells. Since this enhanced glycolysis provides sufficient ATP for trophoblast cells, it may be possible to promote trophoblast proliferation through up-regulation of the cAMP/Rap1/PI3K/AKT pathway. Consistent with this speculation, our proteomic analysis of HLA-F–overexpressing Jar cells revealed a significant upregulation of proteins associated with the PI3K/AKT pathway, including PIK3R1, PIK3CB, AKT1, and AKT2 (Table S3). Although we have not yet validated this potential downstream pathway in HLA-F-overexpressing and knockdown trophoblast cells, the observation that a PKM2 agonist promotes the proliferation of shHLA-F Jar cells in the MiniPDX model suggests that PKM2 itself may serve as a promising therapeutic target for modulating cell proliferation. PKM2 is one of the key enzymes in the glycolysis pathway and is active in either its tetramer or dimer state. Acetylation of PKM2 residue K305 was found to inhibit tetramer formation and thus the activity of this enzyme (Lv et al. 2011). Its specific activators, DASA-58 and TEPP-46, can alleviate the inhibition of enzyme activity due to K305 acetylation and improve tetramer formation (Anastasiou et al. 2012). A recent study reported that the lactylation of PKM2 residue K62 can promote enzyme activity and the differentiation of anti-inflammatory M2 macrophages (Wang et al. 2022). In addition, the ratio of cytoplasmic-to-nuclear expression of PKM2 was found to be positively correlated with enzyme activity (Wang et al. 2022; Alquraishi et al. 2019). PKM2 can promote the invasion function of SW71 trophoblast cells, and treatment with a PKM2 agonist in IUGR animal models can increase placental weight and fetal mouse weight (Tsai et al. 2021). However, the effect of PKM2 agonists on the proliferation of trophoblast cells has not yet been studied, and the potential therapeutic use of PKM2 agonists to address abnormalities in glycolysis—with implications for disorders like preeclampsia—also remains to be explored. As a first step in this direction, this study used a MiniPDX model to confirm the effect of a PKM2 agonist on the proliferation of HLA-F–knockdown trophoblastic cells, and the results suggest that a PKM2 agonist may represent an avenue to explore the treatment of preeclampsia.

Beyond its classic role in antigen presentation, we have, for the first time, demonstrate that HLA-F can bind to the promoter region of PKM2 to promote its transcription and protein translation This finding challenges the conventional understanding of HLA molecules and suggests that their biological functions may be far more complex than previously recognized. However, our study did leave some areas unexplored: for example, we did not examine specific targets for the downstream regulation of trophoblast proliferation by the glycolytic pathway, and we were not able to perform intervention studies of the use of PKM2 agonists on animal models of preeclampsia. The latter is particularly critical, as the efficacy of PKM2 agonists in preeclampsia animal models would provide more clinically relevant insights. Given that glycolysis plays multifaceted roles in biological systems, the impact of PKM2 agonists on tumor growth remains controversial (Singh et al. 2019; Ma et al. 2022). Additionally, it is unclear whether PKM2 agonists can ameliorate key clinical features of preeclampsia, such as hypertension and proteinuria, in mouse models. Another essential next step will be to validate these findings using an HLA-F–knockdown pregnant mouse model. These studies are currently under consideration pending the acquisition of additional funding.

In summary, this study presents preliminary evidence that insufficient HLA-F expression in trophoblastic cells leads to a decrease in glycolysis and abnormal trophoblast proliferation by down-regulating the expression and activity of PKM2, ultimately leading to the development of preeclampsia. In addition, this study also reports for the first time that lactylation of PKM2 residue K305 has an inhibitory effect on the activity of this enzyme. In the absence of effective intervention strategies targeting the HLA-F gene-phenotype and expression level, it is possible that PKM2 agonists may promote placenta formation and have potential therapeutic value for preeclampsia.

## Conclusions

The decrease of HLA-F expression in placental trophoblast cells leds to the downregulation of PKM2 transcription and protein expression, resulting in the downregulation of PKM2-mediated glycolysis level and insufficient energy supply. Meanwhile, the relative up-regulated lactylation of PKM2 K305 led to the decrease in enzyme activity, which all led to the downregulation of trophoblast proliferation capacity and involvement in the pathogenesis of preeclampsia.

## Electronic supplementary material

Below is the link to the electronic supplementary material.

Supplementary Material 1

Supplementary Material 2

Supplementary Material 3

Supplementary Material 4

Supplementary Material 5

## Data Availability

No datasets were generated or analysed during the current study.
