# Supplementary material for: HLA-F regulates the proliferation of trophoblast via PKM2-dependent glycolysis in the pathogenesis of preeclampsia
Source: Mol Med. 2025 Apr 18;31:142. doi: 10.1186/s10020-025-01201-w (PMC12008859; doi:10.1186/s10020-025-01201-w)
Supplement: Supplementary file 1 — Supplementary Material 1 [file 10020_2025_1201_MOESM1_ESM.docx]

|  | Forward | Reverse |
| --- | --- | --- |
| GAPDH | TGACTTCAACAGCGACACCCA | CACCCTGTTGCTGTAGCCAAA |
| PKM2 | ACTGGCATCATCTGTACCATTG | AGCCACATTCATTCCAGACTTA |
| PKM Promter | CCAGAAGCTTGCTACACGTC | GAGGTGGGGAAGGGCTAAAG |
| HLA-F | ACGTAGACGACACGCAATTCCTG | ACCCTGTGGTCCACTCCCAATAC |

Table S5: Primer list
